# How Does Epidemic Prevention Training for Pig Breeding Affect Cleaning and Disinfection Procedures Adoption? Evidence from Chinese Pig Farms

**DOI:** 10.3390/vetsci10080516

**Published:** 2023-08-09

**Authors:** Yufan Chen, Rui Xia, Jinghan Ding, Ze Meng, Yuying Liu, Huan Wang

**Affiliations:** College of Management, Sichuan Agricultural University, Chengdu 611130, China; 202003489@stu.sicau.edu.cn (Y.C.); 202003461@stu.sicau.edu.cn (R.X.); 202003490@stu.sicau.edu.cn (J.D.); 202002246@stu.sicau.edu.cn (Z.M.); yuying.liu@sicau.edu.cn (Y.L.)

**Keywords:** ASF, farmers’ behavior, pig farm biosecurity management, biosecurity cognition

## Abstract

**Simple Summary:**

An animal epidemic poses a severe threat to the economic development of a country. Pig farmers are crucial in controlling the spread of viral infections in pigs. As a crucial component of the biosecurity system, selecting proper cleaning and disinfection (C&D) procedures is a dynamic and long-term decision that requires a higher knowledge base among pig farmers. In this study, we collected data from 333 pig farmers in the Sichuan Province of China—a region significantly affected by the African swine fever (ASF) virus. The collected data were analyzed to elucidate the mechanism of the impact of epidemic prevention training on enhancing pig farmers’ adoption of C&D procedures. The empirical results showed that epidemic prevention training promotes the adoption of C&D procedures among pig farmers. In addition, biosecurity cognition plays a partially mediating role in epidemic prevention training, influencing the adoption of regular and comprehensive C&D procedures.

**Abstract:**

African Swine Fever (ASF) is a highly infectious disease, severely affecting domestic pigs and wild boar. It has significantly contributed to economic losses within the pig farming industry. As a critical component of biosecurity measures, the selection of cleaning and disinfection (C&D) procedures is a dynamic and long-term decision that demands a deeper knowledge base among pig farmers. This study uses a binary logit model to explore the effect of epidemic prevention training on the adoption of C&D procedures among pig farmers with irregular and regular C&D procedures based on micro-survey data obtained from 333 pig farmers from Sichuan. The endogeneity issue was handled using propensity score matching, resulting in solid conclusions. In addition, the critical mediating impact of biosecurity cognition was investigated using a bootstrap analysis. The empirical study demonstrated that epidemic prevention training encourages pig farmers to adopt C&D procedures, with biosecurity cognition significantly mediating. Furthermore, epidemic prevention training was more likely to promote the adoption of C&D procedures among pig farmers with shorter breeding experiences and those having breeding insurance. Our study emphasized the importance of implementing epidemic prevention training to improving pig farmers’ biosecurity cognition and promoting the adoption of C&D procedures. The results included suggested references for preventing ASF and the next epidemic of animal diseases.

## 1. Introduction

African swine fever (ASF) is a highly infectious disease affecting domestic pigs and wild boar [1]. It is distinguished by its high fever and fatality rate [2]. Since 2007, ASF has emerged as a severe threat to the global pork supply, resulting in significant production losses in Mongolia, Vietnam, sub-Saharan Africa, and parts of the European Union [3,4,5]. China is the world’s largest pork producer and consumer [6]. The stability of the Chinese national economy is directly linked to the stability of the hog industry. The first ASF outbreak was reported in the Liaoning Province of China in August 2018. Within eight months, the disease spread to all of China’s mainland provinces and caused a substantial loss to its hog industry [7]. After a peak in the number of outbreaks in the last quarter of 2018, there was an overall decreasing trend in the number of confirmed cases. In the third quarter of 2020, there were only 3 outbreaks, but the ongoing outbreaks continued, and since then, the number of outbreaks has increased again [8]. ASF has resulted in some farmers quitting pig production or scaling back their operations, which restricted pig stocking and restocking and resulted in considerable price fluctuations [9]. Consequently, the Chinese government released policies to guarantee the recovery of pig production. Although research on vaccine development is ongoing, neither a vaccine nor treatment is currently available for ASF, increasing the challenge of controlling it [10,11]. Therefore, prevention and control are the most effective methods of mitigating the adverse impacts of ASF on the global pig population [12,13]. As a result, China introduced the Biosecurity Law of the People’s Republic of China to strengthen the biosecurity system to prevent the ravages of ASF and better cope with the next epidemic.

In the face of the risk of ASF or other pig diseases, prevention must be prioritized by enhancing the hygiene management of pig farms and cutting off the transmission routes of diseases. Therefore, pig farms need to establish a biosecurity barrier to reduce the transmission of pathogens from the outside to the farm and prevent the spread of pathogens within the farm. Cleaning and disinfection (C&D) plays a key role in the control of disease on farms [13]. Pathogenic microorganisms can enter farms via multiple routes. 

Therefore, it is essential to thoroughly cleanse and sanitize all movable elements to minimize the risk of pathogenic outbreaks. C&D of pig farms is a dynamic and continuous process [14]. Specifically, it can be divided into irregular C&D and regular C&D procedures. Irregular C&D procedures refer to the cleaning and disinfection of personnel and vehicles entering the pig farm [15]. Farmers rely on delivery vehicles to convey feed and pigs. Nonetheless, these vehicles may inadvertently transport viruses due to manure and wheel adhesions acquired while traveling to various swine farms. It is essential to thoroughly clean and disinfect the vehicles from the outside to prevent the dissemination of pathogens within the farm. Similarly, all personnel and visitors accessing the production area must adhere to strict C&D procedures. At a minimum, personnel should don work clothes, boots, and mittens that have been disinfected [16]. Personnel are required to adhere to proper personal decontamination and doffing procedures before leaving an infected premise or any quarantined area [13]. This preventive measure is essential in curbing the spread of pathogens. Regular C&D procedures refer to the cleaning and disinfection of buildings and equipment in pig farms at specified periods, including the ceiling, walls, floor, pipelines, feeding troughs, drinking nipples, and other equipment [17]. Regular C&D procedures are more demanding than irregular C&D procedures. This is because regular C&D procedures follow specific steps to ensure each step is performed effectively and efficiently the first time to reduce the need for repeating the process [18]. Farmers should first clean all portable equipment removing all the visible organic matter. The surface should be dried quickly after cleaning. Managers should check the standard of cleaning using a powerful torch and moist white wipes. Finally, farmers should use appropriate disinfectants to disinfect equipment and buildings. Regular C&D of premises must be conducted using the “all in/all out” system between each batch of pigs, followed by a 10-day resting period between batches to maintain low infection rates [13,19]. Although thorough C&D provides for the prevention and control of ASF on pig farms, it places higher demands on the knowledge, attitude, and cognition of pig farmers [20]. How to enhance farmers’ comprehension of C&D to encourage implementation has become a new challenge.

Epidemic prevention training for pig breeding aims to offer pig farmers technical guidance in preventing and controlling pig diseases, thus enhancing their capacity to prevent and control them. In China, training for pig disease prevention and control is frequently conducted during the fall and winter months. This timing aligns with the seasonal nature of disease occurrence in pig herds, as the high incidence of pig diseases typically spans from mid-September to the end of March the following year. Epidemic prevention training provides an excellent solution to encourage pig farmers to adopt C&D procedures. First, knowledge of biosecurity is a crucial driver in influencing behavior [21,22]. Epidemic prevention training increases the level of knowledge and awareness of pig farmers about the best C&D procedures. Second, because pig farmers share information and experience exchange behaviors in social networks, epidemic prevention training can transmit information on disease prevention, control, and C&D procedures to pig farmers through social networks, thus increasing social influence and peer pressure and inspiring pig farmers to adopt C&D procedures more actively. More importantly, epidemic prevention training can provide corresponding management support and follow-up inspection, correct problems and deficiencies in time, improve the training content, and increase pig farmers’ trust and recognition of C&D procedures [23]. However, certain researchers point out that only a few farmers would adopt the good practices taught to them after a training session over the long term [24]. The inadequacy of training methods, lack of capacity, cognitive bias, and social environment contribute to its occurrence. So, how exactly does epidemic prevention affect farmers’ C&D management? Although, numerous studies have examined the effects of personal characteristics, the farming environment, and the policy environment on farmers’ C&D management behaviors [25,26,27,28]. Studies on the influence of epidemic prevention training on farmers’ adoption of C&D procedures from a micro perspective are scarce. Based on these factors, we examined the effects of epidemic prevention training on pig farmers’ implementation of C&D procedures from micro-survey data from the province of Sichuan, analyzing the mediating role of biosecurity cognition in the influence process. Compared with previous studies, the prime innovations of the study are as follows. First, micro-survey data collected from the Sichuan province in 2021 were used to explore the influence of epidemic prevention training on adopting C&D procedures, enriching the research on the impact of epidemic prevention training on adopting biosecurity measures. Second, a Bootstrap mediation analysis model was used to examine the mediating role of farmers’ biosecurity cognition in the impact of epidemic prevention training on the correct implementation of C&D procedures under the impact of ASF. Third, this study explores the effective ways for farmers to strengthen pig farm cleaning and disinfection management under the influence of ASF and provide theoretical and empirical evidence for the government to construct the epidemic prevention training system and improve the biosecurity level of pig farms.

## 2. Materials and Methods

### 2.1. Theoretical Analysis and Research Assumptions

Schultz’s theory of human capital emphasizes training as an essential method to accumulate human capital [29]. Training is pivotal in motivating and empowering individuals to engage in self-directed learning, accumulate practical experience, and significantly enhance their capacity for creativity, knowledge acquisition, and experiential knowledge accumulation [30]. First, epidemic prevention training can not only improve the skills of the trainees, but also enhance the efficiency of using technology by the trainees. Therefore, epidemic prevention training for pig farmers can improve their human capital for efficiency and proficiency in C&D procedures. Second, pig farmers need help transitioning from irregular C&D to regular C&D procedures in practice: higher complexity and learning cost. These factors hinder the proliferation of standardized C&D procedures among pig farmers. Epidemic prevention training can optimize the knowledge structure of pig farmers about farm C&D procedures by disseminating information and providing organized regulation (that is, regulations and restrictions on organizations holding the epidemic prevention training) and improving pig farmers’ awareness of C&D procedures to achieve human capital accumulation. Workers with rich human capital could adjust and correct their early production experience and breeding habits, adopt regular C&D procedures for pig farms, and improve the efficiency of C&D through continuous learning to achieve the synergistic goal of ensuring biosecurity and stable economic benefits. Third, pig farmers not only seek to maximize income, but also lower risk distribution and higher survival security [31]. Risk perception is affected by the knowledge endowment of the pig farmer—that is, the human capital of the pig farmer. It is difficult for pig farmers, who make independent business decisions, to measure the unpredictable risk of the epidemic. Epidemic prevention training can enrich pig farmers’ biosecurity knowledge and make them aware of the importance of using irregular and regular C&D procedures to reduce the risk of ASF introduction. Therefore, they select more comprehensive preventive measures to deal with the risk.

Based on this, the following hypotheses are proposed:

**H1.**  *Epidemic prevention training encourages pig farmers to adopt C&D procedures.*

**H1a.**  *Epidemic prevention training encourages pig farmers to adopt irregular C&D procedures.*

**H1b.**  *Epidemic prevention training encourages pig farmers to adopt regular C&D procedures.*

**H1c.**  *Epidemic prevention training encourages pig farmers to simultaneously adopt irregular and regular C&D procedures.*

Pig farmers’ biosecurity cognition significantly influences their decision-making regarding epidemic prevention and control behavior [32]. Epidemic prevention training can promote the adoption of C&D procedures by improving pig farmers’ biosecurity cognition. First, more advanced epidemic prevention measures have higher requirements for human capital. Unskilled epidemic prevention behaviors could cause economic losses to pig farmers. Thus, improving pig farmers’ understanding of biosecurity management will strengthen knowledge accumulation, improve the level of technology used by pig farmers, and increase the possibility of pig farmers adopting C&D procedures. Second, the subjective cognition of risk for pig farmers is an essential factor affecting their risk management strategies [33]. Failure to conduct scientific and proper C&D procedures by pig farmers could lead to the further spread of swine fever, resulting in substantial economic losses. However, if farmers gain awareness of the risks, it becomes easier to comprehend the potential risks associated with not implementing proper cleaning and disinfection measures. Therefore, strengthening the cognition of pig farmers’ biosecurity can enhance their knowledge of possible disease risks and improve the adoption of C&D procedures.

**H2.**  *Biosecurity cognition plays an important mediating role in epidemic prevention training influencing the adoption of C&D procedures by pig farmers.*

**Figure 1:**
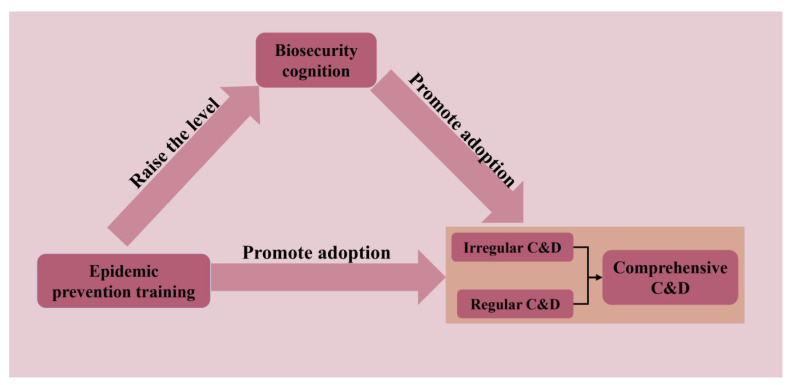
Theoretical model.

Figure 1 shows the theoretical model of this study.

### 2.2. Data Collection

The pig industry constitutes China’s most considerable breeding industry. The risk of the spread of significant epidemic diseases is an important issue faced by China’s pig industry. Pig farmers play an important role and have strong representativeness in epidemic disease prevention and control behaviors adopted by pig farmers and, thus, can better reflect the implementation of animal husbandry disease prevention and control work.

The data were derived from the field research conducted by the research group in Sichuan, China, from July to September 2021. We designed a questionnaire based on the Ministry of Agriculture and Rural Affairs document “Technical Guidelines for the Normalization of African Swine Fever Prevention and Control (Trial Version)”, the local standard “DB51/T 2684-2020 Technical Specifications for the Prevention and Control of African Swine Fever”, and related research literature. The questionnaire included three aspects—namely, personal endowment, epidemic prevention measures, and epidemic prevention cognition, primarily covering personal endowment, basic characteristics of the family, system construction, staffing, organizational guarantee, and other aspects of pig farmers. The research group conducted a pre-survey in Chengdu, Sichuan, and improved the questionnaire according to the pre-research sample data. Afterwards, 20 researchers were recruited, and a 1-week research training was performed; the training content primarily covered questionnaire settings, inquiry methods, and precautions.

The researchers could not perform the study on the pig farm because of the limits imposed by the real production situation of the pig industry. Government restrictions for the prevention of epidemics prevent gathering the management staff of each pig farm, thereby making the research substantially more difficult. Local epidemic prevention personnel led the survey for this project to ensure the representativeness and typicality of the survey and acquire multiple samples under the prevailing conditions. Under the official authorization of the district animal husbandry bureaus, the investigators were guided by the epidemic prevention personnel to visit the pig farm. Face-to-face interviews with the respondents were conducted on the outskirts of the farm premises. The research group employed a sampling approach that combined purposive, progressive, and random sampling methods based on local economic development, pig production, and other relevant factors. The research sample selection adhered to the principles of scientific rigor, accessibility, and diversity. The specific operation process was as follows:

First, the sample province was determined. Sichuan Province is located in the hinterland of Southwest China. It is a predominantly agricultural province. Sichuan’s pig slaughter ranks first in China and is strongly represented. Thus, Sichuan, China, was selected as the research area for this project.

Second, the sample areas were determined. According to the economic development status and development plan of the Sichuan Province, it can be divided into five major regions, among which Chengdu Plain Economic Zone, Sichuan South Economic Zone, and Sichuan Northeast Economic Zone are the leading pig production areas. Based on the allocation table of incentive funds for hog transfer out of large counties in 2021 issued by the Ministry of Agriculture and Rural Development, we selected large hog transfer out counties in Chengdu Plain Economic Zone, Sichuan South Economic Zone, and Sichuan Northeast Economic Zone as sample counties. The large hog transfer-out counties indicated a large local hog breeding scale, a better-developed breeding industry, and an increased sample representative.

**Figure 2:**
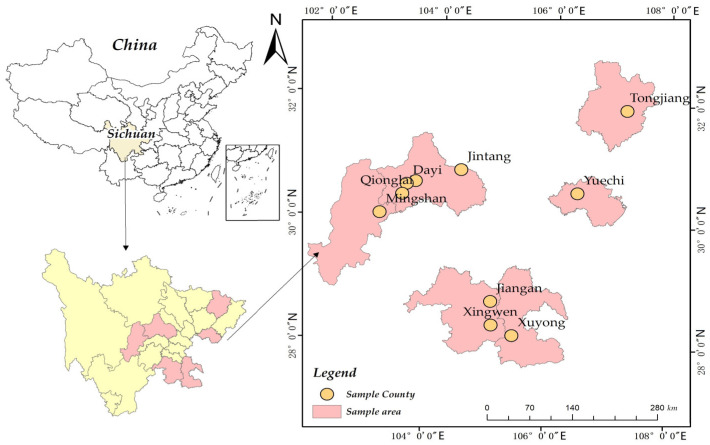
Distribution of sample counties.

Third, sample villages were identified. According to the pig output volume in each county, we chose five counties or county-level cities from the Chengdu Plain Economic Zone and five counties or county-level cities from both the South Sichuan Economic Zone and the Northeast Sichuan Economic Zone. Then, two to five sample townships were identified. Finally, one to three sample villages were selected in each sample township (See Figure 2). Based on the pig farmers list, 5 to 20 farmers were randomly selected from each sample village as subjects. The survey was conducted using one-on-one, face-to-face interviews. All investigators underwent a week of training before the start of the formal survey to ensure that the investigators had a good understanding of the questionnaire. Finally, 351 questionnaires were distributed and 333 valid questionnaires were recovered, with a questionnaire recovery rate of 94.87%.

Pig farmers interviewed had an annual slaughter scale of over 30 pigs, with income from pig farming constituting over 30% of their total annual income. The sample selection largely considered two aspects: First, the breeding scale of free-range pig farmers was relatively small, the proportion of breeding income to the total household income was low, pig breeding could not be the primary business, and there were no primary conditions for the prevention and control of significant epidemics. Second, large-scale pig farmers constitute the backbone of China’s pig breeding industry and the core responsible body for preventing and controlling significant epidemics. Large-scale pig farmers accept most of the relevant technical specifications, standards, and regulatory policies promulgated by the state. Accordingly, we focused on the “backbone” of pig production when selecting samples. It ensured that the research results represented the basic situation of major epidemic prevention and control of pig farmers across China.

### 2.3. Variable Selection

#### 2.3.1. Dependent Variables

We investigated the effects of epidemic prevention training on farmers’ adoption of C&D procedures. Therefore, the dependent variable was whether or not to adopt C&D procedures. C&D involves two aspects. First, when people or vehicles enter the pig farm, they should be cleaned and disinfected. This type of C&D procedure is not regular. Thus, it was defined as an irregular C&D procedure. The value was 1 if the farmer adopted irregular C&D procedures, and 0 otherwise. Second, pig farms should constantly clean and disinfect farms to avoid disease growth and transmission. Regular C&D procedures require farmers to clean and disinfect the barn with pigs once a week under normal circumstances. When there is a threat of a disease epidemic on the farm, the frequency of C&D procedures is increased to twice or thrice a week. If the farm has pigs with ASF, it should be cleaned and disinfected three to five times a day for 7 days, followed by once daily for 15 days. We defined it as the regular C&D procedure. Not all pig farmers adopt irregular and regular C&D procedures simultaneously. Some may adopt only one due to limited resources and perceived limitations. Thus, we employed the comprehensive C&D variable to evaluate whether pig farmers adopt both irregular and regular C&D procedures. To operationalize this variable, a value of 1 was assigned if the farmer implemented both measures and 0 if the farmer implemented only one or none of them, based on the definition of regular and irregular C&D procedures described above.

#### 2.3.2. Independent Variable

The core explanatory variable in this study was epidemic prevention training. In response to the question “Do you participate in epidemic prevention training?”, if the farmer marked “yes”, a value of 1 was assigned; if he received instructions in epidemic prevention and marked a “no”, a value of 0 was assigned. The content of the epidemic prevention training primarily involved characteristics, the current situation, hazards, points for prevention, and prevention methods on five aspects of different major pig diseases, including ASF, foot-and-mouth disease, and blue ear disease. During the survey, we particularly emphasized whether the epidemic prevention training involved standardized procedures for C&D and the selection of disinfectants.

#### 2.3.3. Mediation Variable

We selected biosecurity cognition as our mediation variable based on the previous analyses [6,34,35]. It is described as the degree of a farmer’s knowledge of the principles or requirements of biosecurity management (1–5 increments).

#### 2.3.4. Control Variables

Individual characteristics of pig farmers include gender, age, education, and household labor, which were significantly related to the adoption of epidemic prevention measures by pig farmers [20]. Regarding production and operation characteristics, pig farmers with experience of longer breeding years are more likely to implement changes in their management systems [36]. A larger breeding scale is related to more special assets for pig breeding and a stronger sense of risk avoidance [37]. Similarly, a higher proportion of breeding income is related to the increased dependence of pig farmers’ household income on farming and increased willingness to invest more resources to improve the levels of biosecurity [38]. Therefore, we selected breeding year, breeding scale, and proportion of breeding income as the most basic variables in the characteristics of production and operation and included them in the control variables. In addition, breeding environment characteristics are important influencing factors affecting the participation of pig farmers in epidemic prevention training and the adoption of C&D procedures. Breeding environment characteristics primarily include three aspects—namely, breeding organization, breeding insurance, and government inspection [39,40,41,42]. Therefore, we focused on these three dimensions: “Are you a member of a breeding organization?”, “Do you buy breeding insurance?”, and “Does the government conduct inspections of your farm?”. Control variables were included for assessment. Table 1 presents the variable definitions and descriptions.

### 2.4. Research Methods and Models

#### 2.4.1. Logit Model

After adding control variables, we selected the adoption of C&D procedures as the dependent variable. Epidemic prevention training was taken as the independent variable. The logit model was used to determine the relationship between independent and dependent variables.

(1) $$logit\left(p\right)=\ln\left(\frac{p}{1-p}\right)=\beta_{0}+\beta_{1}X_{1}+\cdots +\beta_{i}X_{i}+\varepsilon_{1}$$

Here, p represents the probability of pig farmers adopting C&D procedures; $X_{1}\ldots$ $\beta_{i}$ $X_{i}$ refers to the independent variable, encompassing the core independent variable and control variables; $\beta_{1}\ldots$ $\beta_{i}$ represents the regression coefficient; $\beta_{0}$ represents the constant term; $\varepsilon_{1}$ signifies the residual term.

#### 2.4.2. Propensity Score Matching Method (PSM Model)

This study focused on whether epidemic prevention training promoted the adoption of C&D procedures, which can be achieved by comparing farmers’ adaptive C&D behavior decisions under the two conditions of participation in epidemic prevention training and non-participation. However, farmers’ responses to adaptive C&D behavior may be self-selected, and different family resource endowments may also affect farmers’ behaviors, leading to selection bias. At the same time, because it is hard to observe the impact of epidemic prevention training on pig farmers when they do not implement C&D measures to cope with ASF, they can only observe the current behavior of farmers to cope with ASF, and the absence of observation data will lead to deviation and biased estimation of samples. Therefore, we used propensity score matching (PSM) to solve this problem and improve the robustness of the results. PSM error correction divided the pig farmers into an experimental group and a control group based on whether they received epidemic prevention training and afterward matched in a certain manner such that the external control conditions remained the same. The impact of epidemic prevention training on adopting C&D procedures in the experimental and control groups was studied by assessing the difference between them to adopt C&D procedures. The specific operation process was as follows. First, the logit regression model was used to calculate the propensity score. Second, according to the score, the experimental and control groups were matched using a suitable algorithm. Finally, the average adaptation willingness (ATT) of the experimental and control groups to adopt C&D procedures was calculated:

(2) $$ATT=E\left[\left(y_{1i}-y_{0i}\right)|D_{i}=1\right]=E\left(y_{1i}|D_{i}=1\right)-E(y_{0i}|D_{i}=1)$$

where $D_{i}$ is a binary variable and i indicates that pig farmers participate in epidemic prevention training. $D_{i}=1$ indicates that the pig farmers participate in epidemic prevention training; otherwise, they are not involved in training. $y_{1i}$ and $y_{0i}$ represent estimates for experimental and control groups, respectively. ATT indicated that pig farmers who participated in epidemic prevention training had adopted C&D procedures at the level $E\left(y_{1i}|D_{i}=1\right),$ and pig farmers who did not participate in epidemic prevention training adopted C&D procedures at the level $E(y_{0i}|D_{i}=1)$ gap. As $E(y_{0i}|D_{i}=1)$ could not be observed, PSM algorithmically placed $E(y_{0i}|D_{i}=1)$ to substitute $E(y_{0i}|D_{i}=0)$.

#### 2.4.3. Mediation Effect Test Model of Bootstrap Method

We used the Bootstrap method [43] for the mediation effect test. Compared with the causal stepwise regression method commonly used in academia [44], the Bootstrap method has improved in terms of testing the rationality of the procedure (the existence of the mediation effect does not need to be significant over), the depth of the test analysis (certain intermediaries imply that there is still an intermediary path that has not been revealed) and the validity of the test method (the Bootstrap method can reduce the probability of making a type of error). Based on this, a specific model was designed as follows:

(3) $$Y=i+cX+e_{1}$$

(4) $$M=i+aX+e_{2}$$

(5) $$Y=i+cX+bM+e_{3}$$

The model considers Y as the adoption of C&D for dependent variables, X as the core independent variable of epidemic prevention training, and M as the mediating variable representing biosecurity cognition. The specific mediation effect test procedure was as follows: the first step tested whether a.b was significant. If a.b was significant—that is, the mediation test result does not contain 0 under the 95% confidence interval—the intermediary path existed. The second step tested if a.b was insignificant—that is, the mediating test result contained 0 under the 95% confidence interval—the intermediary path did not exist, and we skipped to the third step. The third step was to test whether $c^{\prime }$ was significant. If $c^{\prime }$ was insignificant, it was a complete mediating effect; if $c^{\prime }$ was significant, it was a partial mediating effect. In the third step, if a.b was insignificant, the intermediary was not established, and we continued to test whether $c^{\prime }$ was significant. Here, i is the intercept term, a, b, c are the estimation parameters, respectively, and $e_{1}$, $e_{2}$, $\mathrm{and}\;e_{3}$ are residual items of the regression model.

## 3. Results

### 3.1. Descriptive Analysis

The study sample demonstrated that the pig farmers primarily included males, who accounted for 75.68% of the total sample. Individuals aged 40–59 accounted for 72.07 percent of the entire group. This distribution aligned with the prevailing demographic characteristics of pig farming in China, where most pig farming entities were middle-aged males. Additionally, the breeding scale of 30–99 accounted for 61.56% of the sample, while the breeding scale of 100–499 represented 33.33%. This distribution aligned with the prevailing situation of relatively small-sized pig farmers in China. More than half of the total sample relied on pig farming for over 75% of their income, and all participants had more than 30% of their income from farming activities. This indicated that the survey sample adequately represented farmers for whom pig farming is the primary source of livelihood. The basic distributional characteristics of the survey sample are listed in Table 2.

### 3.2. Binary Logit Model Estimation

In this study, we conducted a binary logit regression model of the epidemic prevention training’s impact on pig farmers’ adoption of C&D procedures using the Stata software. Regression coefficients and marginal effects were both presented. As shown in Table 3, epidemic prevention training positively affected pig farmers’ adoption of irregular and regular C&D procedures separately at the 1% and 5% significance levels. It also affected adopting comprehensive C&D procedures with a 1% significance level. For every 1% increase in epidemic prevention training, the probability of pig farmers adopting irregular, regular, and comprehensive C&D procedures increased by 0.133, 0.115, and 0.188 percentage points. However, because the decision of pig farmers to participate in epidemic prevention training is not random, factors such as personal endowment and the external environment could influence the choice of pig farmers, leading to self-selection bias. Therefore, we adopted PSM to avoid the problem of self-selection bias.

### 3.3. Estimation Results of PSM

**Figure 3:**
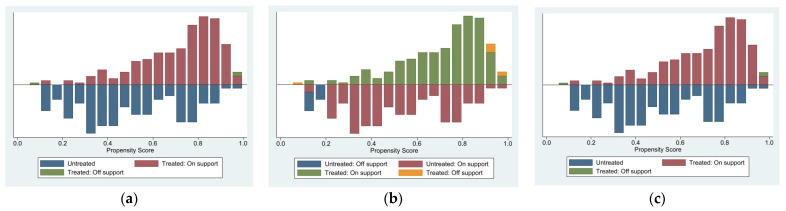
Influence of cohort effect after bias correction. (**a**) Nearest neighbor matching. (**b**) Radius matching. (**c**) Kernel-based matching.

We adopted nearest neighbor matching, radius matching, and kernel matching for estimation. The treatment variable was whether it was for participation in epidemic prevention training, and the matching was based on the propensity score generated from logit regression. Before computing the average treatment effect, it was essential to evaluate using the common support domain and balance test. The specific tests included the following:

This study examined the degree of overlap between the propensity score distribution intervals of the treatment and control groups to ensure matching quality. The common value range of all matching results is presented in Figure 3. Nearest neighbor matching and kernel matching had the most observations located in the common value range, and the matching process only lost three samples, which was an excellent matching effect. In comparison, radius matching lost 16 samples throughout the matching process.

The primary purpose of the homogeneity test was to examine whether the distribution of explanatory variables in the treatment and control groups was well balanced before and after matching. A standard deviation of less than 10% indicated an excellent matching effect. The results clearly showed that the differences in pseudo-R2 values, LR statistics, and standardized deviations of the three matching methods were significantly lower after matching (Table 4). In addition, the standardized deviations were less than 10%, indicating an excellent matching effect, and cleared the balance test.

Table 5 demonstrates the average treatment effect of epidemic prevention training on adopting C&D procedures among pig farmers. We used the bootstrap self-help method with 500 cycles, and the corresponding self-help standard errors were calculated.

With the adoption of irregular C&D procedures, all matching results passed the significance test. The ATT value produced by the nearest neighbor matching was the highest—i.e., 0.183 (Table 5). The results demonstrated that epidemic prevention training facilitated the implementation of irregular C&D procedures. All ATT values for regular C&D procedures were considered positive and cleared the test for significance at a 5% level. The highest ATT value was 0.206 for the nearest neighbor matching, whereas 0.180 was the ATT value for radius matching (Table 5). The results suggested that epidemic prevention training can enhance the adoption of regular C&D procedures among pig farmers. Furthermore, epidemic prevention training had a statistically significant impact on promoting farmers to adopt irregular and regular C&D procedures simultaneously across all three matched outcomes at a level of significance of 1%.

### 3.4. Analysis of Mediating Effects

The Bootstrap method was used to test the mediation functions of biosecurity cognition. The mediating effect was assessed based on whether the results of the mediation test contained 0 at the 95% confidence interval.

As shown in Table 6, the primary effect interval for the effect of epidemic prevention training on the adoption of irregular C&D procedures contained 0. In contrast, the confidence interval for the mediating effect did not contain 0. These results indicated that epidemic prevention training directly affected the adoption of irregular C&D procedures, whereas the impact of biosecurity cognition was insignificant. However, the primary effect interval of the epidemic prevention training on adopting regular C&D procedures did not contain 0, whereas the confidence interval of the mediating effect contains 0. This suggested that epidemic prevention training increased the adoption of regular C&D procedures by increasing the biosecurity cognition of pig farmers. Regular C&D procedures can be lengthy, costly, and involve complex strategies, which may require pig farmers to develop a deeper understanding of biosecurity. Noteworthy, epidemic prevention training directly impacted pig farmers’ adoption of comprehensive C&D procedures and facilitated adoption by enhancing their biosecurity cognition, with both direct and indirect effects having intervals inclusive of 0.

### 3.5. Heterogeneity Analysis

Heterogeneity among pig farmers was one of the most significant internal environmental factors limiting the improvement of pig farms’ biosecurity. The effect of epidemic prevention training on the adoption of C&D procedures with different characteristics can vary. This study examined the impact of epidemic prevention training on the adoption of C&D procedures from two distinct perspectives—namely, breeding years and breeding insurance. The adoption levels of C&D procedures among pig farmers from several heterogeneity perspectives were determined using kernel matching. The findings are presented in Table 7.

On the one hand, the study’s findings indicated that epidemic prevention training significantly promoted regular and comprehensive C&D procedure adoption among pig farmers with shorter breeding experience, at a significant level of 5% and 1%. Conversely, the impact of epidemic prevention training was limited for pig farmers with more ample farming experience. This was because experienced pig farmers relied on their existing knowledge and were less open to adopting new C&D procedures. On the other hand, the adoption of both irregular and regular C&D procedures was significantly promoted by epidemic prevention training among pig farmers who had breeding insurance at a statistical level of 1%. However, for pig farmers without breeding insurance, epidemic prevention training only promoted adopting irregular C&D procedures. A possible explanation was the difference in risk management awareness and resource allocation points of pig farmers with and without breeding insurance. 

## 4. Discussion

The emergence of highly infectious diseases like ASF poses a significant threat to the global agricultural chain and meat production [6]. ASF is primarily facilitated through unsterilized vehicles and people, contributing to 46% of the total transmission [45]. Hence, stringent measures are imperative to clean and disinfect incoming vehicles and personnel. Moreover, the ASF virus exhibits remarkable resilience in protein-rich environments, enabling its survival for extended periods [46]. This extreme environmental resistance is pivotal in the virus‘s localized persistence and geographic spread [47]. Consequently, regular and thorough cleaning and disinfection protocols in pig farms assume paramount importance. In this study, we used the IPWRA model, Bootstrap method, and microdata to evaluate how epidemic prevention training for pig breeding affects pig farmers‘ adoption of C&D procedures. Based on the results, resources and effort should be placed on strengthening epidemic prevention training to improve the management of C&D on pig farms.

According to the results, epidemic prevention training significantly promoted more comprehensive C&D management among pig farmers. Previous studies have consistently demonstrated that biosecurity knowledge drives behavioral changes [21,22]. The lack of such knowledge, coupled with high-risk practices and non-compliance with regulations, contributes to the persistent occurrence of ASF [48]. Training is an interactive learning process that equips pig farmers with essential skills, knowledge, and attitudes while facilitating knowledge-sharing [20]. Farmers are encouraged to effectively adopt various measures to prevent ASF by internalizing and implementing the knowledge gained. Building upon previous studies, our study further investigated the impact of epidemic prevention training on C&D procedures. Epidemic prevention training generates favorable external conditions for farmers to acquire knowledge of C&D procedures. On the one hand, it reduces the perceived complexity of C&D procedures and increases the incentives for pig farmers to improve farm biosecurity. Epidemic prevention training introduces pig farmers to and provides consultations on using C&D procedures, which will be translated into practical behavior when farmers learn the essential biosecurity contents and measures, encouraging the adoption of these procedures. On the other hand, pig producers are informed about the importance of farm biosecurity system construction. Throughout the training, pig farmers comprehensively understood the concept of irregular C&D and the need to clean and disinfect all areas where pigs, humans, and cars passed to prevent spreading infections to another herd. Concurrently, all-in and all-out production were widely encouraged in China, necessitating more C&D of different herds after breeding in the same barn to prevent the spreading of disease between herds. Thus, pig farmers are more willing to adopt comprehensive C&D procedures after participating in epidemic prevention training.

Furthermore, it has been demonstrated that pig farmers’ biosecurity cognition s mediating in adopting C&D programs due to epidemic prevention training. Lengthy periods of knowledge transfer and technical instruction in epidemic prevention training can increase pig farmers’ knowledge of biosecurity content and measures. It also emphasizes the significance of regular C&D procedures. As farmers’ biosecurity cognition improves, they become more willing to sacrifice economic benefits to manage and reduce farming risks, ultimately leading them to select more comprehensive C&D measures to improve farm biosecurity. Previous research had indicated that production experience is shaped by the accumulation of cognition and knowledge, crucial variables in individual and organizational decision-making [49,50]. These factors significantly influence the behavioral intentions of individuals and ultimately impact their behavioral decisions. Governments need to build trust with pig farmers and make them aware of their critical role in preventing ASF and of the underlying purpose of ASF control and eradication measures so that they adopt a range of biosecurity measures [38,51]. This study contributed to the important role of increased biosecurity cognition among pig farmers for their excellent disease prevention and control.

At the same time, there are differences in the effect of epidemic prevention training on different pig farms due to differences in individual endowments. This study examined that pig farmers who had been in the industry for a more extended period had extraordinary farm management expertise and were more likely to implement advanced biosecurity measures. It was consistent with the previous study [36]. Furthermore, pig farmers with farm insurance were more likely to adopt C&D procedures, enhancing farm biosecurity. Pig farmers having breeding insurance have a greater knowledge of risk management [52]. They were more likely to forgo cost advantages in favor of adopting more comprehensive C&D methods to improve farm biosecurity and lower the risk of pig illnesses. In contrast, non-insured pig farmers tend to expend more effort on irregular C&D procedures because they tend to be more risk-averse and hesitate to invest significantly in biosecurity management [33].

## 5. Conclusions and Policy Recommendations

### 5.1. Conclusions

Because the participation of every pig farmer in biosecurity construction affects the whole country’s pig disease prevention and control strategy, it is crucial to rapidly promote C&D procedures, improve the biosecurity level of pig farms, and enhance the risk prevention capability of pig farms. In this study, based on the data from 333 pig farmers in Sichuan, China, the logit and PSM models were used to estimate the impact of epidemic prevention training on pig farmers’ adoption of C&D procedures, and the Bootstrap method was used to study the mediating role of biosecurity cognition. The results demonstrated that: (a) epidemic prevention training reinforced the adoption of C&D procedures taken by pig farmers. Specifically, epidemic prevention training played a crucial role in promoting the adoption of both irregular and regular C&D procedures by pig farmers and can lead to simultaneous adoption. (b) Epidemic prevention training can encourage pig farmers to adopt regular C&D procedures through biosecurity cognition. (c) Heterogeneity exists among different farming groups. After receiving epidemic prevention training, younger pig farmers and those with breeding insurance will likely adopt more comprehensive C&D procedures.

### 5.2. Policy Recommendations

First, biosecurity involves the public interest of the whole society and is difficult to address by grassroots governance effectively. Given the positive impact of epidemic prevention training on pig farmers’ adoption of C&D procedures, the government should take the construction of an epidemic prevention training system as an essential path to enhance the biosecurity level of pig farming and support it in terms of financial, material, and human resources. In this endeavor, the government should lead in establishing a comprehensive training system for pig epidemic prevention, fostering active involvement from pig breeding enterprises, scientific research institutes, and breeding associations.

Second, bolster pig farmers’ biosecurity cognition through their proactive participation. Utilizing participatory training as a bridge strengthens local pig farmers’ communication and makes them better understand the realities of pig epidemics, promoting cross-learning and collaboration. Furthermore, regularly evaluating the effectiveness of the training and seeking feedback from participants empowers them to actively contribute to developing epidemic prevention training elements. Through risk communication and community involvement, pig farmers’ biosecurity cognition is continually improved. Third, it needs to tailor training programs designed for various generational groups to address the heterogeneity among different pig farmers. Additionally, the curriculum of epidemic prevention training is continuously enriched and innovated in line with the advancements in pig epidemic prevention technology and methodologies. Moreover, the government should provide breeding insurance subsidies to pig farmers to enhance their ability to cope with epidemic risks. Breeding insurance subsidies can avoid the loss of rent-seeking behavior associated with direct financial contributions and improve management efficiency.

## 6. Limitations and Future Research Direction

This study had certain limitations, which could result in research gaps. First, this study focused solely on the effect of epidemic prevention training approaches on adopting C&D procedures in pig farms. Future studies can investigate the influence mechanisms of training in different methods of epidemic prevention. Second, we examined the value of C&D procedures from two perspectives: irregular C&D and regular C&D procedures in pig farms. However, C&D is a systematic endeavor that treats waste and disinfects inputs. In this regard, future research can focus on multiple facets of C&D in pig farms. Increasing the degree of biosecurity on pig farms entails several interdependent elements. Moreover, we examined the farmers’ adoption of C&D procedures from a social science perspective and did not measure the effectiveness of C&D procedures. Although farmers adopted C&D procedures, their effectiveness could be affected by different procedures and different chemical concentrations of disinfecting agents. This aspect is yet to be explored in-depth and could be a future research focus.

## Figures and Tables

**Table 1:** Variable definitions and descriptive statistics.

Variables	Variable Measure	Mean	S.D.
Irregular C&D procedures	Do you require incoming personnel and vehicles to be cleaned and disinfected? (0 = no, 1 = yes)	0.766	0.424
Regular C&D procedures	Do you regularly clean and disinfect the pig farm in compliance with the regulations? (0 = no, 1 = yes)	0.718	0.451
Comprehensive C&D procedures	Do you take both regular and irregular disinfection measures? (0 = no, 1 = yes)	0.538	0.499
Epidemic prevention training	Did you participate in the epidemic prevention training this year? (0 = no, 1 = yes)	0.637	0.482
Gender	Gender of respondents (Female = 0; Male = 1)	0.757	0.430
Age	Age of respondents (year)	48.171	9.942
Education	Primary school and below = 1; Junior high school = 2; Senior high school/technical secondary school = 3; Junior college = 4; Undergraduate and above = 5	1.778	0.832
Family labor	How many people are involved in pig farming in the household?	1.646	0.769
Breeding year	5 years and below =1; 6–10 = 2; 11–15 = 3; 16–20 = 4; 21 and above = 5	2.919	1.381
Breeding scale	30–99 = 1; 100–499 = 2; 500–999 = 3; 1000–1999 = 4; 2000 and above = 5	1.492	0.786
Proportion of breeding income	30–49% = 1; 50–74% = 2; 75% and above = 3	2.324	0.766
Breeding organization	Are you a member of a breeding organization? (such as cooperative, company) (0 = no, 1 = yes)	0.105	0.307
Breeding insurance	Do you buy breeding insurance? (0 = no, 1 = yes)	0.727	0.446
Government inspections	Does the government conduct inspections of your farm? (0 = no, 1 = yes)	0.628	0.484
Biosecurity cognition	Your level of knowledge of the contents or requirements of farm biosecurity management (1–5 increments)	3.240	1.447

**Table 2:** Basic distributional characteristics of the survey sample.

Variables	Classification	Sample Size	Proportion (%)	Variables	Classification	Sample Size	Proportion (%)
Gender	Male Female	252 81	75.68 24.32	Government inspections	Yes No	209 124	62.76 37.24
Age	Under 30 years old 30–39 years old 40–49 years old 50–59 years old Over 59 years old	20 46 109 131 27	6.01 13.81 32.73 39.34 8.11	Breeding scale	30–99 100–499 500–999 1000–1999 2000 and above	205 111 4 7 6	61.56 33.33 1.20 2.10 1.80
Education	Primary school and below Junior high school Senior high school/technical secondary school Junior college Undergraduate and above	143 136 41 11 2	42.94 40.84 12.31 3.30 0.60	Breeding year	5 years and below 6–10 11–15 16–20 21 and above	71 59 88 56 59	21.32 17.72 26.43 16.82 17.72
Proportion of breeding income	30–49% 50–74% 75% and above	61 103 169	18.32 30.93 50.75	Family labor	1 2 3 and above	161 141 31	48.35 42.34 9.31
Breeding organization	Yes No	35 298	10.51 89.49	Breeding insurance	Yes No	242 91	72.67 27.33

**Table 3:** Regression results of the binary logit model.

Variables	Irregular C&D Procedures		Regular C&D Procedures		Comprehensive C&D Procedures	
	Coefficient	Dy/dx	Coefficient	Dy/dx	Coefficient	Dy/dx
Epidemic prevention training	0.788 *** (0.298)	0.133 *** (0.049)	0.754 ** (0.314)	0.115 ** (0.046)	0.949 *** (0.280)	0.188 *** (0.052)
Gender	0.0680 (0.321)	0.012 (0.054)	0.378 (0.330)	0.057 (0.050)	0.555 * (0.304)	0.110 * (0.059)
Age	−0.0327 ** (0.0161)	−0.006 ** (0.003)	0.027 (0.0169)	0.004 (0.003)	−0.009 (0.015)	−0.00186 (0.003)
Education	−0.238 (0.185)	−0.040 (0.031)	0.226 (0.217)	0.034 (0.033)	−0.087 (0.177)	−0.0172 (0.035)
Family labor	−0.0373 (0.183)	−0.006 (0.031)	0.338 (0.224)	0.051 (0.034)	0.281 (0.176)	0.0558 (0.034)
Breeding year	0.144 (0.107)	0.024 (0.018)	0.261 ** (0.116)	0.040 ** (0.017)	0.232 ** (0.010)	0.0460 ** (0.019)
Breeding scale	0.328 (0.221)	0.056 (0.037)	0.859 *** (0.320)	0.131 *** (0.047)	0.547 ** (0.220)	0.108 ** (0.042)
Proportion of breeding income	−0.285 (0.201)	−0.048 (0.034)	0.018 (0.202)	0.003 (0.031)	−0.206 (0.180)	−0.0408 (0.035)
Organization	0.0994 (0.501)	0.017 (0.085)	1.946 * (1.068)	0.296 * (0.161)	0.575 (0.484)	0.114 (0.095)
Insurance	−0.087 (0.321)	−0.015 (0.054)	−0.218 (0.334)	−0.033 (0.051)	0.031 (0.300)	0.006 (0.060)
Government inspections	0.034 (0.300)	0.006 (0.051)	1.236 *** (0.305)	0.188 *** (0.042)	0.829 *** (0.271)	0.165 *** (0.051)
_cons	2.560 ** (1.044)		−4.55 *** (1.190)		−2.312 ** (0.994)	
N	333		333		333	
Chi^2^	18.62 *		87.67 ***		73.03 ***	
Pseudo R^2^	0.051		0.221		0.159	

Note: Standard errors are shown in parentheses. * *p* < 0.1, ** *p* < 0.05, *** *p* < 0.01.

**Table 4:** Equilibrium test results.

Matching Algorithms	Item	Epidemic Prevention Training	
		Unmatched	Matched
Nearest neighbor matching (1:2)	Pseudo-R^2^	0.176	0.048
	LR statistics	76.90	27.75
	MeanBias	31.8	9.8
Radius matching (caliper 0.02)	Pseudo-R^2^	0.176	0.034
	LR Statistics	76.90	19.56
	MeanBias	31.8	8.4
Kernel-based matching (bandwidth 0.06)	Pseudo-R^2^	0.176	0.037
	LR Statistics	76.90	21.38
	MeanBias	31.8	9.2

**Table 5:** Average treatment effects of different matching algorithms.

Matching Algorithms	Irregular C&D Procedures		Regular C&D Procedures		Comprehensive C&D Procedures	
	ATT	T-value	ATT	T-value	ATT	T-value
Nearest neighbor matching (1:2)	0.183 **	2.36	0.206 **	2.58	0.278 ***	3.48
	(0.089)		(0.091)		(0.091)	
Radius matching (caliper 0.02)	0.172 **	2.35	0.180 **	2.36	0.269 ***	3.55
	(0.075)		(0.079)		(0.083)	
Kernel-based matching (bandwidth 0.06)	0.176 **	2.48	0.175 **	2.36	0.262 ***	3.54
	(0.076)		(0.074)		(0.074)	

Note: Standard errors are shown in parentheses. ** *p* < 0.05, *** *p* < 0.01.

**Table 6:** Mediation analysis.

	Irregular C&D Procedures			Regular C&D Procedures			Comprehensive C&D Procedures		
	Coefficient	95% Confidence Interval		Coefficient	95% Confidence Interval		Coefficient	95% Confidence Interval	
		Lower-Bound	Upper-Bound		Lower-Bound	Upper-Bound		Lower-Bound	Upper-Bound
Direct effects	0.138 **	0.025	0.251	0.096 *	−0.002	0.194	0.173 ***	0.054	0.293
	(0.058)			(0.054)			(0.061)		
Indirect effects	0.004	−0.013	0.022	0.030 **	0.003	0.057	0.029 **	0.001	0.057
	(0.009)			(0.014)			(0.014)		

Note: Standard errors are shown in parentheses. * *p* < 0.1, ** *p* < 0.05, *** *p* < 0.01.

**Table 7:** Heterogeneity analysis.

Variables	Irregular C&D Procedures		Regular C&D Procedures		Comprehensive C&D Procedures	
	ATT	T-value	ATT	T-value	ATT	T-value
Shorter breeding years (10 years and below)	0.142	1.15	0.296 **	2.31	0.321 ***	2.83
	(0.131)		(0.127)		(0.126)	
Longer breeding years (Over 10 years)	0.193 *	1.83	0.108	1.05	0.219 *	1.95
	(0.109)		(0.103)		(0.119)	
No breeding insurance	0.400 * (0.213)	2.54	−0.045 (0.175)	−0.45	0.247 (0.209)	1.41
Having breeding insurance	0.128 (0.096)	1.52	0.194 ** (0.088)	2.22	0.257 *** (0.093)	2.87

Note: Standard errors are shown in parentheses. * *p* < 0.1, ** *p* < 0.05, *** *p* < 0.01.

## Data Availability

The data presented in this study are available within the article.

## References

[1] Sánchez-Vizcaíno J.M., Mur L., Martínez-López B. (2012). African Swine Fever: An Epidemiological Update. Transbound. Emerg. Dis..

[2] Costard S., Mur L., Lubroth J., Sanchez-Vizcaino J.M., Pfeiffer D.U. (2013). Epidemiology of African Swine Fever Virus. Virus Res..

[3] Nguyen-thi T., Pham-thi-ngoc L., Nguyen-ngoc Q., Dang-xuan S. (2021). An Assessment of the Economic Impacts of the 2019 African Swine Fever Outbreaks in Vietnam. Front. Vet. Sci..

[4] Ebwanga E.J., Ghogomu S.M., Paeshuyse J. (2022). Molecular Characterization of ASFV and Differential Diagnosis of Erysipelothrix in ASFV-Infected Pigs in Pig Production Regions in Cameroon. Vet. Sci..

[5] Lamberga K., Oļševskis E., Seržants M., Bērziņš A., Viltrop A., Depner K. (2020). African Swine Fever in Two Large Commercial Pig Farms in LATVIA—Estimation of the High Risk Period and Virus Spread within the Farm. Vet. Sci..

[6] Xu G., Sarkar A., Qian L., Shuxia Z., Rahman A., Yongfeng T. (2022). The Impact of the Epidemic Experience on the Recovery of Production of Pig Farmers after the Outbreak-Evidence from the Impact of African Swine Fever (ASF) in Chinese Pig Farming. Prev. Vet. Med..

[7] Tian X., von Cramon-Taubadel S. (2020). Economic Consequences of African Swine Fever. Nat. Food.

[8] Ito S., Bosch J., Martínez-Avilés M., Sánchez-Vizcaíno J.M. (2022). The Evolution of African Swine Fever in China: A Global Threat?. Front. Vet. Sci..

[9] Ma M., Wang H.H., Hua Y., Qin F., Yang J. (2021). African Swine Fever in China: Impacts, Responses, and Policy Implications. Food Policy.

[10] Mutua F., Dione M. (2021). The Context of Application of Biosecurity for Control of African Swine Fever in Smallholder Pig Systems: Current Gaps and Recommendations. Front. Vet. Sci..

[11] Penrith M.L. (2020). Current Status of African Swine Fever. CABI Agric. Biosci..

[12] Domenech J., Lubroth J., Eddi C., Martin V., Roger F. (2006). Regional and International Approaches on Prevention and Control of Animal Transboundary and Emerging Diseases. Ann. N. Y. Acad. Sci..

[13] De Lorenzi G., Borella L., Alborali G.L., Prodanov-Radulović J., Štukelj M., Bellini S. (2020). African Swine Fever: A Review of Cleaning and Disinfection Procedures in Commercial Pig Holdings. Res. Vet. Sci..

[14] Gosling R. (2018). A Review of Cleaning and Disinfection Studies in Farming Environments. Farm Pract..

[15] Kousta M., Mataragas M., Skandamis P., Drosinos E.H. (2010). Prevalence and Sources of Cheese Contamination with Pathogens at Farm and Processing Levels. Food Control.

[16] Ford W.B. (1995). Disinfection Procedures for Personnel and Vehicles Entering and Leaving Contaminated Premises. Rev. Sci. Tech..

[17] Pletinckx L.J., Dewulf J., De Bleecker Y., Rasschaert G., Goddeeris B.M., De Man I. (2013). Effect of a Disinfection Strategy on the Methicillin-resistant Staphylococcus Aureus CC398 Prevalence of Sows, Their Piglets and the Barn Environment. J. Appl. Microbiol..

[18] Štukelj M., Prodanov-Radulović J., Bellini S. (2021). Cleaning and Disinfection in the Domestic Pig Sector. Understanding and Combatting African Swine Fever: A European Perspective.

[19] Luyckx K., Millet S., Van Weyenberg S., Herman L., Heyndrickx M., Dewulf J., De Reu K. (2016). A 10-Day Vacancy Period after Cleaning and Disinfection Has No Effect on the Bacterial Load in Pig Nursery Units. BMC Vet. Res..

[20] Dione M.M., Dohoo I., Ndiwa N., Poole J., Ouma E., Amia W.C., Wieland B. (2020). Impact of Participatory Training of Smallholder Pig Farmers on Knowledge, Attitudes and Practices Regarding Biosecurity for the Control of African Swine Fever in Uganda. Transbound. Emerg. Dis..

[21] Young J.R., Evans-Kocinski S., Bush R.D., Windsor P.A. (2015). Improving Smallholder Farmer Biosecurity in the Mekong Region Through Change Management. Transbound. Emerg. Dis..

[22] Cui B., Liu Z.P. (2016). Determinants of Knowledge and Biosecurity Preventive Behaviors for Highly Pathogenic Avian Influenza Risk Among Chinese Poultry Farmers. Avian Dis..

[23] Muriithi B., Bundi M., Galata A., Miringu G., Wandera E., Kathiiko C., Odoyo E., Kamemba M., Amukoye E., Huqa S. (2018). Biosafety and Biosecurity Capacity Building: Insights from Implementation of the NUITM-KEMRI Biosafety Training Model. Trop. Med. Health.

[24] Dione M., Ouma E., Opio F., Kawuma B., Pezo D. (2016). Qualitative Analysis of the Risks and Practices Associated with the Spread of African Swine Fever within the Smallholder Pig Value Chains in Uganda. Prev. Vet. Med..

[25] Kouam M.K., Jacouba M., Moussala J.O. (2020). Management and Biosecurity Practices on Pig Farms in the Western Highlands of Cameroon (Central Africa). Vet. Med. Sci..

[26] Ribbens S., Dewulf J., Koenen F., Mintiens K., De Sadeleer L., de Kruif A., Maes D. (2008). A Survey on Biosecurity and Management Practices in Belgian Pig Herds. Prev. Vet. Med..

[27] Kouam M.K., Moussala J.O. (2018). Assessment of Factors Influencing the Implementation of Biosecurity Measures on Pig Farms in the Western Highlands of Cameroon (Central Africa). Vet. Med. Int..

[28] Niemi J.K., Sahlström L., Kyyrö J., Lyytikäinen T., Sinisalo A. (2016). Farm Characteristics and Perceptions Regarding Costs Contribute to the Adoption of Biosecurity in Finnish Pig and Cattle Farms. Rev. Agric. Food Environ. Stud..

[29] Schultz T.W. (1961). Investment in Human Capital. Am. Econ. Rev..

[30] Arokiasamy L., Fujikawa T., Piaralal S.K., Arumugam T. (2023). A Systematic Review of Literature on Human Capital Investment and Its Significance for Human Resource Development. Int. J. Syst. Assur. Eng. Manag..

[31] Cui B., Liu Z.P., Ke J., Tian Y. (2019). Determinants of Highly Pathogenic Avian Influenza Outbreak Information Sources, Risk Perception and Adoption of Biosecurity Behaviors among Poultry Farmers in China. Prev. Vet. Med..

[32] Bora M., Bora D.P., Manu M., Barman N.N., Dutta L.J., Kumar P.P., Poovathikkal S., Suresh K.P., Nimmanapalli R. (2020). Assessment of Risk Factors of African Swine Fever in India: Perspectives on Future Outbreaks and Control Strategies. Pathogens.

[33] Valeeva N.I., van Asseldonk M.A.P.M., Backus G.B.C. (2011). Perceived Risk and Strategy Efficacy as Motivators of Risk Management Strategy Adoption to Prevent Animal Diseases in Pig Farming. Prev. Vet. Med..

[34] Chenais E., Boqvist S., Sternberg-Lewerin S., Emanuelson U., Ouma E., Dione M., Aliro T., Crafoord F., Masembe C., Ståhl K. (2017). Knowledge, Attitudes and Practices Related to African Swine Fever Within Smallholder Pig Production in Northern Uganda. Transbound. Emerg. Dis..

[35] Omitoyin S.A., Osakuade K.D. (2021). Awareness and Constraints of Aquaculture Biosecurity Among Fish Farmers in Ekiti State, Nigeria. Aquac. Stud..

[36] Toma L., Stott A.W., Heffernan C., Ringrose S., Gunn G.J. (2013). Determinants of Biosecurity Behaviour of British Cattle and Sheep Farmers-A Behavioural Economics Analysis. Prev. Vet. Med..

[37] Simon-Grifé M., Martín-Valls G.E., Vilar-Ares M.J., García-Bocanegra I., Martín M., Mateu E., Casal J. (2013). Biosecurity Practices in Spanish Pig Herds: Perceptions of Farmers and Veterinarians of the Most Important Biosecurity Measures. Prev. Vet. Med..

[38] Xu B., Zhou L., Qiu C., Li Y., Zhang W. (2021). What Determines Pig Farmers’ Epidemic Coping Behaviors: A Qualitative Analysis of Endemically Infected Areas in Relation to African Swine Fever. Vet. Sci..

[39] Nantima N., Davies J., Dione M., Ocaido M., Okoth E., Mugisha A., Bishop R. (2016). Enhancing Knowledge and Awareness of Biosecurity Practices for Control of African Swine Fever among Smallholder Pig Farmers in Four Districts along the Kenya–Uganda Border. Trop. Anim. Health Prod..

[40] Tanquilut N.C., Espaldon M.V.O., Eslava D.F., Ancog R.C., Medina C.D.R., Paraso M.G.V., Domingo R.D. (2020). Biosecurity Assessment of Layer Farms in Central Luzon, Philippines. Prev. Vet. Med..

[41] Schemann K., Taylor M.R., Toribio J.A.L.M.L., Dhand N.K. (2011). Horse Owners’ Biosecurity Practices Following the First Equine Influenza Outbreak in Australia. Prev. Vet. Med..

[42] Cooper T.L., Smith D., Gonzales M.J.C., Maghanay M.T., Sanderson S., Cornejo M.R.J.C., Pineda L.L., Sagun R.A.A., Salvacion O.P. (2022). Beyond Numbers: Determining the Socioeconomic and Livelihood Impacts of African Swine Fever and Its Control in the Philippines. Front. Vet. Sci..

[43] Preacher K.J., Rucker D.D., Hayes A.F. (2007). Addressing Moderated Mediation Hypotheses: Theory, Methods, and Prescriptions. Multivar. Behav. Res..

[44] Baron R.M., Kenny D.A. (1986). The Moderator-Mediator Variable Distinction in Social Psychological Research: Conceptual, Strategic, and Statistical Considerations. J. Pers. Soc. Psychol..

[45] Dixon L.K., Sun H., Roberts H. (2019). African Swine Fever. Antivir. Res..

[46] Geering W.A., Penrith M.L., Nyakahuma D. (2001). Manual on Procedures for Disease Eradication by Stamping Out.

[47] Juszkiewicz M., Walczak M., Woźniakowski G. (2019). Characteristics of Selected Active Substances Used in Disinfectants and Their Virucidal Activity against ASFV. J. Vet. Res..

[48] Dione M.M., Ouma E.A., Roesel K., Kungu J., Lule P., Pezo D. (2014). Participatory Assessment of Animal Health and Husbandry Practices in Smallholder Pig Production Systems in Three High Poverty Districts in Uganda. Prev. Vet. Med..

[49] Randrianantoandro T.N., Kono H., Kubota S. (2015). Knowledge and Behavior in an Animal Disease Outbreak–Evidence from the Item Count Technique in a Case of African Swine Fever in Madagascar. Prev. Vet. Med..

[50] Costard S., Zagmutt F.J., Porphyre T., Pfeiffer D.U. (2015). Small-Scale Pig Farmers’ Behavior, Silent Release of African Swine Fever Virus and Consequences for Disease Spread. Sci. Rep..

[51] Moskalenko L., Schulz K., Mõtus K., Viltrop A. (2022). Pigkeepers’ Knowledge and Perceptions Regarding African Swine Fever and the Control Measures in Estonia. Prev. Vet. Med..

[52] Zhang Y.H., Li C.S., Liu C.C., Chen K.Z. (2013). Prevention of Losses for Hog Farmers in China: Insurance, on-Farm Biosecurity Practices, and Vaccination. Res. Vet. Sci..

